# Provider perspectives on beta-lactam therapeutic drug monitoring programs in the critically ill: a protocol for a multicenter mixed-methods study

**DOI:** 10.1186/s43058-021-00134-9

**Published:** 2021-03-24

**Authors:** Erin F. Barreto, Andrew D. Rule, Mohammad H. Alshaer, Jason A. Roberts, Mohd Hafiz Abdul Aziz, Marc H. Scheetz, Kristin C. Mara, Paul J. Jannetto, Ognjen Gajic, John C. O’Horo, Kasey R. Boehmer

**Affiliations:** 1 Department of Pharmacy, Mayo Clinic, 200 1st St SW, Rochester, MN 55905 USA; 2 Robert D. and Patricia E. Kern Center for the Science of Health Care Delivery, Mayo Clinic, 200 1st St SW, Rochester, MN 55905 USA; 3 Division of Epidemiology, Mayo Clinic, 200 1st St SW, Rochester, MN 55905 USA; 4 Division of Nephrology and Hypertension, Mayo Clinic, 200 1st St SW, Rochester, MN 55905 USA; 5 Infectious Disease Pharmacokinetics Lab, Emerging Pathogens Institute, University of Florida, 1600 SW Archer Rd, Gainesville, FL 32610 USA; 6 Department of Pharmacotherapy and Translational Research, College of Pharmacy, University of Florida, 1600 SW Archer Rd, Gainesville, FL 32610 USA; 7 University of Queensland Centre for Clinical Research (UQCCR), Faculty of Medicine, The University of Queensland, Royal Brisbane and Women’s Hospital, Brisbane, QLD 4029 USA; 8 Department of Pharmacy Practice, Chicago College of Pharmacy, Midwestern University, 555 31st St, Downers Grove, IL 60515 USA; 9 Pharmacometrics Center of Excellence, Midwestern University, 555 31st St, Downers Grove, IL 60515 USA; 10 Division of Biomedical Statistics and Informatics, Mayo Clinic, 200 1st St SW, Rochester, MN 55905 USA; 11 Department of Laboratory Medicine & Pathology, Mayo Clinic, 200 1st St SW, Rochester, MN 55905 USA; 12 Division of Pulmonary and Critical Care Medicine, Mayo Clinic, 200 1st St SW, Rochester, MN 55905 USA; 13 Division of Infectious Diseases, Mayo Clinic, 200 1st St SW, Rochester, MN 55905 USA; 14 Knowledge and Evaluation Research (KER) Unit, Mayo Clinic, 200 1st St SW, Rochester, MN 55905 USA

**Keywords:** Study protocol, Mixed-methods, Implementation, Therapeutic drug monitoring, Beta-lactams, Pharmacokinetics/pharmacodynamics, Intensive care unit

## Abstract

**Background:**

Beta-lactams (i.e., penicillins, cephalosporins, carbapenems, monobactams) are the most widely used class of antibiotics in critically ill patients. There is substantial interpatient variability in beta-lactam pharmacokinetics which renders their effectiveness and safety largely unpredictable. One strategy to ensure achievement of therapeutic concentrations is drug level testing (“therapeutic drug monitoring”; TDM). While studies have suggested promise with beta-lactam TDM, it is not yet widely available or implemented. This protocol presents a mixed-methods study designed to examine healthcare practitioners’ perspectives on the use and implementation of beta-lactam TDM in the critically ill.

**Methods:**

An explanatory sequential mixed-methods design will be used [QUANT → qual]. First, quantitative data will be collected through a web-based questionnaire directed at clinicians at three academic medical centers at different phases of beta-lactam TDM implementation (not yet implemented, partially implemented, fully implemented). The sampling frame will include providers from a variety of disciplines that interact with drug level testing and interpretation in the critical care environment including pharmacists, intensivists, infectious diseases experts, medical/surgical trainees, and advanced practice providers. Second, approximately 30 individuals will be purposively sampled from survey respondents to conduct in-depth qualitative interviews to explain and expand upon the results from the quantitative strand. Normalization Process Theory and the Consolidated Framework for Implementation Science will be used to guide data analysis.

**Discussion:**

These data will be used to answer two specific questions: “What are ICU practitioners’ perspectives on implementing beta-lactam TDM?” and “What factors contribute to the success of beta-lactam TDM program implementation?” Results of this study will be used to design future implementation strategies for beta-lactam TDM programs in the critically ill.

**Trial registration:**

NCT04755777.

**Supplementary Information:**

The online version contains supplementary material available at 10.1186/s43058-021-00134-9.

Contributions to the literature

- Use of implementation science frameworks will evaluate the “work” associated with implementation of beta-lactam therapeutic drug monitoring, and the contextual factors that influence its success.
- Study is situated in the high-risk intensive care unit environment.
- Merging and triangulation of quantitative and qualitative strands will identify areas where participants have similar or dissimilar perceptions or practices.
- Data analysis designed to inform implementation of beta-lactam therapeutic drug monitoring to promote scale and spread.

## Background

Beta-lactams account for at least 70% of the antibiotics utilized in caring for critically ill patients [1]. Inclusive of cephalosporins, penicillins, carbapenems, and monobactams, beta-lactam antibiotics form the backbone of all major treatment algorithms in the critically ill including for undifferentiated sepsis, pneumonia, bacteremia, urinary tract infections, intra-abdominal infections, skin and soft tissue infections, and meningitis [2–6]. Substantial inter- [7, 8] and intra-patient [9] variability in beta-lactam concentrations have been observed in critically ill patients which is thought to contribute to suboptimal effectiveness, safety, and development of antibiotic resistance [10]. For this reason, international guidelines and consensus statements have advocated for the use of novel approaches to personalize beta-lactam therapy including real-time drug level testing (also referred to as “therapeutic drug monitoring” or TDM) [2, 11, 12].

Beta-lactam TDM is designed to optimize the drug level within the therapeutic window to ensure maximal effectiveness and safety. For the beta-lactam antibiotics, achievement of an adequate drug level (fraction of time above the minimum inhibitory concentration of the organism) is associated with a higher likelihood of clinical success [7] and a decrease in the potential for antimicrobial resistance [13]. Conversely, excessive beta-lactam exposure has been associated with an increased risk of concentration-dependent toxicities, most notably neurotoxicity [14–16]. Beta-lactam TDM programs have been used in adult and pediatric critically ill patients. In these studies, when the drug levels were evaluated 2–3 days into therapy (at the assumed pharmacokinetic steady state), 40–90% of patients failed to achieve the desired target beta-lactam levels. The majority of non-target levels were low which underscores the need for therapeutic optimization [17–20].

While TDM for antimicrobials in the critically ill is not new, its application to beta-lactams remains poorly adopted. In a multicenter multinational survey of antimicrobial dosing and monitoring practices in intensive care units (ICUs) in Europe, only approximately 10% of respondents reported use of TDM for piperacillin/tazobactam and carbapenems. When used, it was described as infrequent, and pharmacokinetic/pharmacodynamic targets varied widely across institutions [21]. In a national cross-sectional study about antibiotic TDM practices distributed to physicians in ICUs in Germany, 17% and 22% of respondents reported use of TDM for piperacillin and meropenem [22]. Several possible explanations exist for the limited adoption. One is the relatively limited access to beta-lactam assays (often a send-out test) which results in prolonged turnaround time. Other assays for drugs where TDM is more common are widely available and performed on automated chemistry analyzers with FDA approved assays. There are also limited data that conclusively demonstrate the link between TDM-informed dosing and more favorable clinical outcomes than with standard strategies. Finally, there may be implementation challenges of such a program given the high frequency of beta-lactam use in critically ill patients. TDM has historically been limited to antimicrobials with narrow spectrums of activity (treat only select groups of bacteria), relatively infrequent use, and narrow therapeutic windows (often a high risk for toxicity). Chief among the examples of agents with substantial literature in support of the role for TDM include vancomycin and the aminoglycosides. In the previously mentioned German evaluation, in contrast to the beta-lactams, 75% of ICU practitioners report routine use of TDM for vancomycin [22].

Even with more mainstream antibiotics that undergo TDM, a clear gap in the literature exists surrounding the implementation considerations associated with these TDM programs from the perspective of the end user [23]. Perhaps the closest corollary is the recent effort with vancomycin to characterize implementation of the change from trough- to area under the curve-based monitoring (two different pharmacokinetic targets) [24–26]. Extrapolated from these narrative descriptions, several key factors pertaining to implementation appear important. Implementation includes a series of phases including preparation, go-live, and evaluation, each of which requires careful planning. TDM practices are governed locally by clinical practice groups such as in the intensive care unit, the pharmacy and therapeutics committees, and the antimicrobial stewardship teams. Input from leadership of these groups as well as end-users is necessary to ensure successful implementation. Chief among the factors that appear to most influence implementation of TDM programs is clinician familiarity. Education, training, and real-time support delivered by identified local champions may be used to enhance clinician awareness, knowledge, and confidence. Moreover, proactive attention to logistical issues (e.g., documentation, online resources, electronic health record integration) may facilitate a streamlined and successful implementation experience. Collectively much more information is necessary to better understand how best to efficiently and effectively implement beta-lactam TDM.

This manuscript presents the protocol of a mixed-methods study which aims to characterize the barriers and facilitators for beta-lactam TDM implementation in real-world practice from the perspective of a diverse group of ICU clinicians. At the conclusion of this study, we seek to answer the following two questions:

1. What are ICU practitioners’ perspectives on implementing beta-lactam TDM?
2. What factors contribute to successful implementation of beta-lactam TDM programs?

## Methods/design

### Overall design

**Fig. 1:**
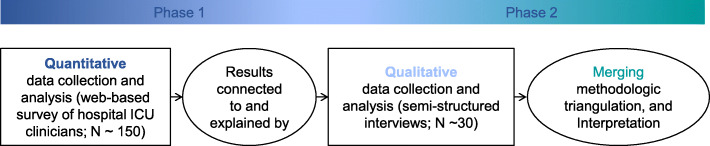
Study schema

This study will use a two-phase explanatory sequential mixed-methods design to evaluate factors which influence beta-lactam TDM in critical care practice (Fig. 1; QUANT → qual). A mixed-methods design was selected to not only measure provider perspectives on the need for testing but to use first-person accounts to describe perceived barriers and facilitators associated with implementation of beta-lactam TDM. The quantitative strand will be the principal strand and the qualitative strand will be the complementary method. The study was ethically approved by the local Institutional Review Boards and ethics committees prior to beginning enrollment and has been registered at ClinicalTrials.gov (NCT04755777). The design and reporting of this study is informed by best practices for survey research [27, 28], qualitative research [29], and mixed-methods studies [30] (Additional file 1).

### Survey development

For the quantitative strand, a web-based survey was designed expressly for this study based on previous evidence in the area [21, 24–26] (Additional file 2) in consultation with the local Survey Research Center. Study data will be collected and managed using Qualtrics. Response options for most survey items are close-ended and used statements of agreement and Likert scales.

Seven clinicians (2 intensivists, 3 critical care pharmacists, 1 infectious diseases/antimicrobial stewardship pharmacist, 1 critical care nurse) from diverse hospitals (community and academic) across the USA not involved with the study reviewed and pre-tested the questionnaire. A structured critique form was given to each of these individuals upon survey completion which included specific probes designed to examine question clarity, response options, missing or superfluous survey items, and overall length. Critiques were reviewed and themes identified. Edits were made to questions and responses to address areas of ambiguity. Suggestions about breadth and depth of content resulted in removal of four survey items and addition of eight others. Of these eight additional, seven were open-ended response options from branching logic (e.g., “Please describe your other goal…”). Mean estimated time to complete the survey in pretesting was 8 min.

### Sample

Data will be obtained from ICU clinicians at three hospitals with distinct beta-lactam dosing and monitoring practices.

*Center 1* is a large tertiary referral hospital in Australia. Beta-lactam TDM began at center 1 in 2009 and is *fully implemented* in the routine care of critically ill patients. The hospital offers a variety of acute care services including an active emergency department, intensive care services for 36-ICU beds, and care for hematology/oncology patients including those undergoing hematopoietic stem cell transplantation. 2.25 full time equivalent pharmacists provide a 7-day per week intensive care service that includes pharmacokinetic monitoring of medications. At the outset of the beta-lactam TDM program, the target drug concentration was based on the total drug level, measured as a trough, which targeted a threshold of 4× the minimum inhibitory concentration (MIC) breakpoint of the known or suspected organism. Currently, Center 1 measures free drug concentrations and targets have been tailored to local MIC distributions. Consistently, beta-lactam TDM is performed in ICU patients but has been extended to other services including the outpatient antimicrobial treatment team. Beta-lactam assays are available once daily Monday through Saturday.

*Center 2* is a large university-affiliated academic medical center in the southeast region of the USA which has been performing beta-lactam TDM since 2016 and currently it is considered *partially implemented*. Center 2 offers a wide array of acute care services and has more than 200 ICU beds including medical, surgical, neurological, cardiac, and thoracic and vascular units. There are 11 ICU pharmacists and 3 infectious diseases specialty pharmacists. The antimicrobial TDM is performed by the physicians and pharmacists. It is currently non-protocolized but encouraged for critically ill patients with suspected or documented infections. The recommended approach to beta-lactam TDM is to order peak (1 h after the end of the infusion) and trough samples (30 min prior to the next dose) for each patient. Most often clinically, however, isolated troughs are collected. Once collected, samples are sent to the local pharmacokinetics laboratory which offers LC-MS/MS assays for 11 beta-lactams. Batched beta-lactam assays are performed once daily Monday through Friday. The results are delivered to the electronic health record (Epic) for clinical interpretation and PK/PD calculations.

*Center 3* is a large integrated academic health system in the Midwest region of the USA. Center 3 has developed and validated LC-MS/MS assays for total concentrations of piperacillin, tazobactam, cefepime, and meropenem, but these are *not yet implemented* or available for clinical care. The center includes two hospital campuses at its Midwest location across which there are more than 200 total ICU beds. TDM for antimicrobials and other agents in the ICU are performed by decentral clinical pharmacists for which there are 42 across the two hospitals. Four infectious disease specialty pharmacists also consult on ICU patients. Laboratory facilities to deliver TDM are staffed 24-h/day, 7 days/week. It is expected that when available clinically, the assays will be batched and performed once daily, 7 days/week.

### Phase 1—quantitative strand

#### Data collection

Data will be sourced from a multicenter cross-sectional survey of clinicians working with critically ill patients at the study sites of interest. Healthcare providers from a variety of disciplines interact with drug level testing and interpretation in the critical care environment. Based on our previous experiences with implementing new antibiotic dosing nomograms [31, 32] and the published literature on TDM [24–26], key clinician stakeholders likely to be involved with beta-lactam TDM in the intensive care unit include pharmacists, intensivists, infectious diseases experts, medical/surgical trainees, and advanced practice providers. These individuals will form the sampling frame for the quantitative strand of the mixed-methods study. The objective will be to reflect insights from a range of practitioners including those with considerable experience with routine beta-lactam TDM to those with little or no exposure to it. It would be expected that all of these individuals would have familiarity with antibiotic TDM, in general, but maybe not specific to this drug class.

The study team involves investigators from each participating site. These individuals will be primarily responsible for identifying study participants. To minimize the potential for bias, the questionnaire will be distributed by a survey center independent of the investigative group. Eligible individuals will be contacted via e-mail and invited to participate in the survey. By following the survey link in the structured electronic communication, clinicians will indicate their consent to participate. Reminder communication will occur electronically at 2 weeks and 3 weeks, and the survey will close at the conclusion of the fourth week. Demographic information will be collected including the clinician’s self-reported role on the care team (attending physician, trainee, advanced practice provider, pharmacist), specialty (ICU vs infectious diseases), and years of post-graduate practice experience. To facilitate purposive sampling for the qualitative strand of the study, individuals will be asked to indicate their willingness to be contacted for a future individual interview.

#### Analysis

Based on the mean number of individuals employed at these institutions in the clinical specialties of interest, we expect to administer the survey to approximately 250 individuals at each site (750 individuals total). At each site, we estimate between 5 and 40 individuals are available for sampling in each of the provider subtypes of interest. Recent estimates indicate that physician response rate to surveys approximates 15–20% depending on survey mode [33]. In previous multicenter studies, we observed a response rate between 22 and 47% influenced by clinician subtype [34, 35]. Therefore, we estimate conservatively that 150 individuals will respond to the web-based survey and be eligible for analysis.

Survey response data will be described with frequencies and percentages, and means with standard deviations. Individuals may answer (or not answer) any question on the survey. Missing data will be analyzed for patterns but will not be imputed. The denominator for each survey item will be described. The Pearson’s chi-square test or Fisher’s exact test will be used to analyze independent binary outcomes stratified according to groups (e.g., clinician subtype, level of experience). Survey response rate and results will be compared between the three sites. A *p*-value < 0.05 will be considered statistically significant for these analyses.

### Phase 1—qualitative strand

#### Data collection

Qualitative data will be used to expand upon and further understand the data from the questionnaire results. Data from the qualitative strand will primarily be obtained from semi-structured interviews or, if needed to accommodate clinician schedules, focus groups of less than five clinicians.

Upon completion of the quantitative strand of the study and preliminary data analysis, individuals will be purposively sampled for the qualitative strand from among those who indicated a willingness to participate in follow-up interviews [30]. Qualitative data collection is expected to occur over 6 months with analysis thereafter. To represent the breadth of potential viewpoints on the topic of beta-lactam TDM, the sample will be selected to achieve maximal diversity. We will include individuals from each center, a variety of clinical disciplines and experience, and a range of perspectives on the need for beta-lactam TDM based on key survey items (Q14, “How many critically ill patients treated with beta-lactams should receive TDM (drug level testing)?”; Q16a, “The current approach to dosing and monitoring beta-lactams is suitable for critically ill patients.”; Q16c, “Beta-lactam TDM is relevant to my current practice.”). Further sampling will be considered if other areas of needed diversity are identified. We aim to include approximately 30 clinician stakeholders. Based on the estimated response rate for the quantitative strand of the study, we expect there will be > 10 individuals in each clinician subgroup eligible for sampling. In previous qualitative work at the study center [36], this sample size has been sufficient to achieve thematic saturation. Eligible individuals will be contacted by a researcher or trained study coordinator to obtain oral consent for participation in the interview. Interviews will be limited to one-time interactions of 30–45 min, in person or over the phone.

Interviews will be facilitated using a semi-structured interview guide (Additional file 3) designed expressly for this study. Prior to beginning the interview, subjects will be asked to provide oral consent. We will probe interviewees about the expected or observed impact of beta-lactam TDM on their daily work, and barriers and facilitators associated with its implementation. To capture insights on both the technical details of beta-lactam TDM and determinants of implementation, the first five interviews will be conducted jointly by two study team members, a pharmacist (EB) and a trained health services researcher. Review of the first set of interviews will be used to refine the interview guide at which point the health services researcher will independently conduct the remaining interviews.

#### Analysis

For the qualitative strand, interview transcripts will be uploaded into NVivo software, a qualitative data analysis tool. NVivo aids investigators by facilitating coding of source data, data sorting, and identification of similarities in coded concepts indicative of themes. A trained health services researcher and the principal investigator will independently inductively code preliminary source data in NVivo. Investigators will meet regularly to discuss codes and develop the study codebook. This step will be repeated until no new codes emerge. These codes along with a priori identified codes related to constructs taken from implementation science theories and frameworks (deductive) will then be applied to all source data. After data have been coded, the two investigators will meet to discuss themes emerging in the data. To enhance the confirmability of the findings, a diverse study team will be engaged to review the coding and we will use reflexivity to understand personal biases of study team members.

### Data analysis and integration

Merging will occur after both quantitative and qualitative data collection and analyses are completed. Data will be integrated from the two strands to identify areas of complementarity, concordance, and discordance. Direct quotes from participants will be used in tables and in the study results to characterize the perspectives of critical care clinicians. The integrated findings will be summarized to develop a conceptual model for implementing beta-lactam TDM in critical care practice.

### Frameworks

Two complementary models grounded in implementation science will be used to guide data analysis: Normalization Process Theory (NPT) [37] and the Consolidated Framework for Implementation Science (CFIR) [38].

The NPT is a framework that describes the “work” people do to implement new processes in healthcare [37]. It includes 4 domains: coherence work, participation work, operational work, and appraisal work (possible examples provided in Table 1). The first two domains (coherence work and participation work) can be applied to each hospital, but operational work and appraisal work are only suitable for data from individuals at centers 1 and 2 where routine beta-lactam TDM has been at least partially implemented.

**Table 1:** Potential “work” associated with beta-lactam TDM categorized according to NPT domains

NPT domain	Example for beta-lactam TDM
**Coherence** Work related to a practice’s meaning, use, or utility	Physicians can distinguish the new approach from their current method of beta-lactam monitoring.
**Participation** Involves initiation, enrollment, and legitimation or “buy-in” among human actors about a practice	Pharmacists agree that beta-lactam TDM should be a part of their work.
**Operational (“Collective action”)** Integration of a practice in a specific context, availability of necessary resources to enact the practice	Beta-lactam TDM is adequately integrated in the electronic health record.
**Appraisal (“Reflexive monitoring”)** Judgments and data related to the effectiveness and utility of a new practice	Team can access information about the results of routine monitoring and impact on clinical outcomes.

*NPT* Normalization Process Theory, *TDM* therapeutic drug monitoring

The CFIR is a frequently used framework to organize and interpret factors which influence implementation of an evidence-based practice [38]. The implementation framework includes five constructs: intervention characteristics, outer setting, inner setting, characteristics of the individuals involved with implementation, and the implementation process (possible examples provided in Table 2). CFIR has been used prior to implementing an intervention to identify implementation barriers and facilitators and select an appropriate implementation strategy [39].

**Table 2:** Potential associations between provided responses and CFIR constructs

CFIR construct	Example for beta-lactam TDM
**Intervention characteristics** The intervention being implemented in an organization	Processes for specimen collection
	Turnaround time
	Results reporting in the electronic health record
**Outer setting** Economic, political, social context within which the organization exists	Statements from professional organizations encouraging beta-lactam TDM (e.g., SCCM, ESICM, ESCMID)
	Global emphasis on personalized medicine
**Inner setting** Structural, political, and cultural contexts through which implementation occurs	Academic environment at the hospital
	Multiple providers from various disciplines caring for the same patient (e.g., ICU, pharmacist, ID)
**Individuals** Individuals involved with implementation	Knowledge about antibiotic TDM
	Belief in the ability to execute beta-lactam TDM in clinical practice
	Early versus late adopter (Rogers Diffusion of Innovation)
**Process** Implementation process	Stakeholder engagement in creation of the workflow
	Development of an implementation toolkit
	Local champions who are early users

*CFIR* Consolidated Framework for Implementation Research, *TDM* therapeutic drug monitoring

The use of both frameworks is necessary as the sample is drawn from centers at very different stages of implementation (from complete implementation to planning for implementation). NPT and CFIR constructs were mapped to questionnaire items and will be integrated throughout data analysis.

## Discussion

This study was developed in response to the call for implementation of beta-lactam TDM for critically ill patients by international working groups [2, 11, 12] and the limited literature to guide the optimal approach. Beta-lactams are used in the majority of critically ill patients. Without a careful approach to implementation, introduction of beta-lactam TDM risks harm, waste, or confusion among clinicians. This study will have several strengths. To the best of our knowledge, this study is the first to describe implementation considerations of beta-lactam TDM. A mixed-methods study grounded in implementation science is ideally situated to provide rich data on this topic. Proactive preparation for implementation has the potential to enhance diffusion of evidence-based practice into clinical care [40]. These data can be used to inform future effectiveness-implementation hybrid clinical trials. Inclusion of academic medical centers at different stages of beta-lactam TDM implementation will provide a breadth and depth of perspectives on the topic.

Potential limitations exist with the proposed study. Although the focus is on implementation in the critical care environment, we are not planning to comprehensively survey all clinicians in that environment at the included centers (e.g., not including nursing staff, respiratory therapists, physical therapists). It is possible that other core groups of clinicians relevant to the study aims may be identified from preliminary data. Should this be the case, we will explore administration of a second survey or round of interviews to capture the insights from this identified group. Future studies would also be needed to explore clinician viewpoints outside of the ICU. The included centers are academic medical centers and insights gained may not be generalizable to a community hospital or rural practice. The quantitative study results could be affected by non-response error which is common in physician surveys [33]. Efforts will be made to minimize the impact of this error including identification of a local study champion, securing pre-emptive buy-in from local leadership, and use of multiple reminders. The response rate will be described and compared to published literature [33–35].

## Conclusions

Beta-lactam utilization is situated in a complex multi-level and multi-dimensional context that needs to be characterized before new TDM programs can be implemented [40, 41]. Perceptions of clinician stakeholders in the ICU will be used to describe enthusiasm for beta-lactam TDM programs, team roles, protocols, technology interfaces, and work flow. These will be modeled using existing theoretical frameworks to optimize potential for successful implementation in future trials and clinical practice.

## Supplementary Information

**Additional file 1.** Standardized reporting checklist.

**Additional file 2.** Survey instrument.

**Additional file 3.** Semi-structured interview guide.

## Data Availability

Not applicable.

## Abbreviations

TDM: Therapeutic drug monitoring
ICUs: Intensive care units
MIC: Minimum inhibitory concentration
LC-MS/MS: Liquid chromatography, tandem mass spectrometry
NPT: Normalization Process Theory
CFIR: Consolidated Framework for Implementation Research
