# Supplementary material for: Provider perspectives on beta-lactam therapeutic drug monitoring programs in the critically ill: a protocol for a multicenter mixed-methods study
Source: Implement Sci Commun. 2021 Mar 24;2:34. doi: 10.1186/s43058-021-00134-9 (PMC7992791; doi:10.1186/s43058-021-00134-9)
Supplement: Supplementary file 1 — Additional file 1. Standardized reporting checklist. [file 43058_2021_134_MOESM1_ESM.pdf]

Table 1 Consolidated criteria for reporting qualitative studies (COREQ): 32-item checklist

No	Item	Guide questions/description
**Domain 1: Research team and reflexivity**		
Personal Characteristics		
1.	Interviewer/facilitator	Which author/s conducted the interview or focus group? page 13
2.	Credentials	What were the researcher's credentials? *E.g. PhD, MD* page 13
3.	Occupation	What was their occupation at the time of the study? page 13
4.	Gender	Was the researcher male or female? page 13
5.	Experience and training	What experience or training did the researcher have? page 13
Relationship with participants		
6.	Relationship established	Was a relationship established prior to study commencement? page 13
7.	Participant knowledge of the interviewer	What did the participants know about the researcher? e.g. *personal goals, reasons for doing the research* page 13, additional files 1 & 2
8.	Interviewer characteristics	What characteristics were reported about the interviewer/facilitator? e.g. *Bias, assumptions, reasons and interests in the research topic* page 13, additional files 1 & 2
**Domain 2: study design**		
Theoretical framework		
9.	Methodological orientation and Theory	What methodological orientation was stated to underpin the study? *e.g. grounded theory, discourse analysis, ethnography, phenomenology, content analysis* page 14
Participant selection		
10.	Sampling	How were participants selected? *e.g. purposive, convenience, consecutive, snowball* page 12
11.	Method of approach	How were participants approached? e.g. *face-to-face, telephone, mail, email* page 13
12.	Sample size	How many participants were in the study? pages 11, 13
13.	Non-participation	How many people refused to participate or dropped out? Reasons? pages 11, 13
Setting		
14.	Setting of data collection	Where was the data collected? e.g. *home, clinic, workplace* page 13
15.	Presence of non-participants	Was anyone else present besides the participants and researchers? page 13
16.	Description of sample	What are the important characteristics of the sample? *e.g. demographic data, date* additional file 1
Data collection		
17.	Interview guide	Were questions, prompts, guides provided by the authors? Was it pilot tested? page 8 page 13, addl. file 2.
18.	Repeat interviews	Were repeat interviews carried out? If yes, how many? N/A
19.	Audio/visual recording	Did the research use audio or visual recording to collect the data? page 13
20.	Field notes	Were field notes made during and/or after the interview or focus group? N/A
21.	Duration	What was the duration of the interviews or focus group? page 13
22.	Data saturation	Was data saturation discussed? page 13
23.	Transcripts returned	Were transcripts returned to participants for comment and/or correction? N/A
**Domain 3: analysis and findingsz**		
Data analysis		
24.	Number of data coders	How many data coders coded the data? page 13
25.	Description of the coding tree	Did authors provide a description of the coding tree? page 13
26.	Derivation of themes	Were themes identified in advance or derived from the data? pages 13-15.
27.	Software	What software, if applicable, was used to manage the data? page 13
28.	Participant checking	Did participants provide feedback on the findings? N/A
Reporting		
29.	Quotations presented	Were participant quotations presented to illustrate the themes / findings? Was each quotation identified? e.g. *participant number* page 14
30.	Data and findings consistent	Was there consistency between the data presented and the findings? N/A
31.	Clarity of major themes	Were major themes clearly presented in the findings? page 14
32.	Clarity of minor themes	Is there a description of diverse cases or discussion of minor themes? page 14.

(ii) Participant selection: Researchers should report how participants were selected. Usually purposive sampling is used which involves selecting participants who share particular characteristics and have the potential to provide rich, relevant and diverse data pertinent to the research question [13, 17]. Convenience sampling is less optimal because it may fail to capture important perspectives from difficult-to-reach people [16]. Rigorous attempts to recruit participants and reasons for non-participation should be stated to reduce the likelihood of making unsupported statements [18].

Downloaded from https://academic.oup.com/intqhc/article-abstract/19/6/349/1791966 by Mayo Clinic, Division of Biostatistics user on 14 June 2020
