# Supplementary material for: Provider perspectives on beta-lactam therapeutic drug monitoring programs in the critically ill: a protocol for a multicenter mixed-methods study
Source: Implement Sci Commun. 2021 Mar 24;2:34. doi: 10.1186/s43058-021-00134-9 (PMC7992791; doi:10.1186/s43058-021-00134-9)
Supplement: Supplementary file 2 — Additional file 2. Survey instrument. [file 43058_2021_134_MOESM2_ESM.docx]

Start of Block: Intro

Q1
             


 Introduction
 
 
 
 Thank you for agreeing to participate in this questionnaire.  The objective is to understand your practice and perceptions about the use of beta-lactam therapeutic drug monitoring the critically ill.  The focus of this survey is on the use of therapeutic drug monitoring in the context of infection treatment (not prophylaxis).   For the purpose of this questionnaire, therapeutic drug monitoring or what will be abbreviated as ‘TDM,’ is the use of drug levels to assess whether a patient is achieving their treatment goals.  Commonly in practice we use therapeutic drug monitoring for other antibiotics like vancomycin and aminoglycosides.  In this study we are seeking to understand its application to beta-lactams such as cephalosporins, penicillins, and carbapenems.

| 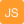 |
| --- |

Q2
**Select your primary practice site.**

- Mayo Clinic (1)
- Royal Brisbane and Women’s Hospital (2)
- UF Health Shands (3)

| Page Break |  |
| --- | --- |

| 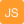 |
| --- |

Q3 **Select your role on the healthcare team.**

- Pharmacist (1)
- Advanced practice provider (i.e., nurse practitioner, physician assistant) (2)
- Physician (resident/fellow) (3)
- Physician (attending) (4)
- Other (5)

Display This Question:

If Q3 = 5

Q4 **Please describe your other role on the healthcare team.**

________________________________________________________________

| 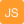 |
| --- |

Q5 **Indicate below if you do NOT want to be contacted for a follow-up interview.** (You will still have an opportunity to decline participation in the interview at the time of contact if you choose.)

- I do NOT want to be contacted for a follow-up interview. (1)

End of Block: Intro

Start of Block: TDM Practice

| 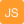 | 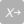 |
| --- | --- |

Q6 Therapeutic Drug Monitoring (TDM) Practice
 
 
 
**In the last 6 months, have you been involved with beta-lactam therapeutic drug monitoring (TDM; drug level testing) as part of your routine patient care activities?**

- Yes (1)
- No (0)

Display This Question:

If Q6 = 1

| 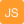 | 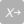 |
| --- | --- |

Q7 **In the last 1 month, please estimate the number of patients for whom you have used beta-lactam TDM (drug level testing) to guide care for infected critically ill patients.**

- 0 (0)
- 1 to 10 (1)
- 11 to 20 (2)
- 21 to 30 (3)
- Greater than 30 (4)

End of Block: TDM Practice

Start of Block: Provider – Perceptions

| 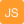 |
| --- |

Q8 Provider Perceptions


**How burdensome is beta-lactam TDM (drug level testing) for infected critically ill patients in an average day’s workload?** If not currently available at your center, describe your perception in anticipation of its availability.

- Very burdensome (1)
- Somewhat burdensome (2)
- Limitedly burdensome (3)
- Not at all burdensome (4)

Q9 **Which of the following are the primary potential barriers to implementation of beta-lactam TDM (drug level testing) in your opinion?** If not currently available at your center, describe your perception in anticipation of its availability. (Select all that apply.)

- Clinician unfamiliarity (1)
- Not enough time in the work day (2)
- Turnaround time (3)
- Cost of testing (4)
- Amount of training needed (5)
- Unclear benefits (6)
- Other (7)

Display This Question:

If Q9 = 7

Q10 **Please describe the other barrier(s) to implementation of beta-lactam TDM (drug level testing) you identify.**

________________________________________________________________

| 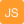 |
| --- |

Q11 **Mark your level of agreement with the following statement: I would like education on beta-lactam TDM (drug level testing).**

- Strongly agree (1)
- Agree (2)
- No opinion/unsure (3)
- Disagree (4)
- Strongly disagree (5)

| Page Break |  |
| --- | --- |

| 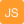 |
| --- |

Q12
**Select which healthcare team member should be primarily responsible for managing beta-lactam TDM (drug level testing) in infected critically ill patients.**

- Any pharmacist (1)
- Pharmacist for the ICU (2)
- Infectious diseases/antimicrobial stewardship pharmacist (3)
- Infectious diseases/antimicrobial stewardship provider (e.g., physician, advanced practice provider) (4)
- Nurse (5)
- Intensivist (6)
- Other critical care provider (resident, fellow, advanced practice provider) (7)
- Other (8)

Display This Question:

If Q12 = 8

Q13 **Please describe the other healthcare team member that should be primarily responsible for managing beta-lactam TDM (drug level testing) in critically ill patients.**

________________________________________________________________

| 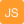 |
| --- |

Q14 **How many critically ill patients treated with beta-lactams should receive TDM (drug level testing)?**

- All (1)
- Most (2)
- Some (3)
- Few (4)
- None (5)

Q15 **Describe which critically ill patients should receive beta-lactam TDM (drug level testing) in your opinion.**

________________________________________________________________

________________________________________________________________

________________________________________________________________

________________________________________________________________

________________________________________________________________

| Page Break |  |
| --- | --- |

| 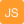 |
| --- |

Q16 **Please indicate to what extent you agree or disagree with each of the following statements:**

|  | Strongly agree (1) | Agree (2) | No opinion/Unsure (3) | Disagree (4) | Strongly disagree (5) |
| --- | --- | --- | --- | --- | --- |
| The current approach to dosing and monitoring beta-lactams at my institution is suitable for infected critically ill patients. (1) |  |  |  |  |  |
| Beta-lactam TDM (drug level testing) is relevant to my current practice. (2) |  |  |  |  |  |
| Beta-lactam TDM (drug level testing) improves treatment effectiveness. (3) |  |  |  |  |  |
| Beta-lactam TDM (drug level testing) decreases antibiotic resistance. (4) |  |  |  |  |  |
| Beta-lactam TDM (drug level testing) improves treatment safety. (5) |  |  |  |  |  |
| The benefits of beta-lactam TDM (drug level testing) for infected critically ill patients outweigh the costs. (6) |  |  |  |  |  |
| Q6 = 1  Turnaround time for beta-lactam drug level reporting at my center is adequate. (7) |  |  |  |  |  |
| Q6 = 1  Beta-lactam TDM (drug level testing) is adequately integrated in the electronic health record. (8) |  |  |  |  |  |
| The proportion of beta-lactam levels within the target range in my work unit is reported (e.g., you can find information about the percent achievement in semi-real time or on a monthly or yearly basis). (9) |  |  |  |  |  |
| I feel comfortable recommending beta-lactam TDM (drug level testing) for infected critically ill patients. If not currently available at your center, describe your comfort in anticipation of its availability. (10) |  |  |  |  |  |
| I can accurately apply the results of a beta-lactam drug level test to make treatment decisions for infected critically ill patients. (11) |  |  |  |  |  |
| I feel comfortable with the preferred target range for beta-lactam drug levels in infected critically ill patients. (12) |  |  |  |  |  |

Display This Question:

If Q16 = 3 [ 1 ]

Or Q16 = 3 [ 2 ]

Q17 **In your opinion, how does beta-lactam TDM (drug level testing) improve treatment effectiveness?**

________________________________________________________________

________________________________________________________________

________________________________________________________________

________________________________________________________________

________________________________________________________________

Display This Question:

If Q16 = 4 [ 1 ]

Or Q16 = 4 [ 2 ]

Q18 **In your opinion, how does beta-lactam TDM (drug level testing) decrease antibiotic resistance?**

________________________________________________________________

________________________________________________________________

________________________________________________________________

________________________________________________________________

________________________________________________________________

Display This Question:

If Q16 = 5 [ 1 ]

Or Q16 = 5 [ 2 ]

Q19 **In your opinion, how does beta-lactam TDM (drug level testing) improve treatment safety?**

________________________________________________________________

________________________________________________________________

________________________________________________________________

________________________________________________________________

________________________________________________________________

| Page Break |  |
| --- | --- |

| 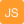 |
| --- |

Q20 **The preferred LOWER limit of the beta-lactam target range in infected critically ill patients is…** (MIC: Minimum inhibitory concentration)

- I don't know. (1)
- 50% of time above MIC during the dosing interval (2)
- 100% of time above MIC during the dosing interval (3)
- 50% of the time above 4x the MIC during the dosing interval (4)
- 100% of time above 4x the MIC during the dosing interval (5)
- I use a specific drug level number (6)
- Other (7)

Display This Question:

If Q20 = 6

| 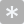 |
| --- |

Q21 **Please list the drug level number you target for the LOWER limit of the CEFEPIME target range (mg/L).**

________________________________________________________________

Display This Question:

If Q20 = 6

| 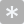 |
| --- |

Q22 **Please list the drug level number you target for the LOWER limit of the PIPERACILLIN target range (mg/L).**

________________________________________________________________

Display This Question:

If Q20 = 6

| 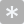 |
| --- |

Q23 **Please list the drug level number you target for the LOWER limit of the MEROPENEM target range (mg/L).**

________________________________________________________________

Display This Question:

If Q20 = 7

| 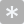 |
| --- |

Q24 **Please list the other LOWER limit of the beta-lactam target range.**

________________________________________________________________

| Page Break |  |
| --- | --- |

| 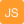 |
| --- |

Q25 **The preferred UPPER limit of the beta-lactam target range in infected critically ill patients is…** (MIC: Minimum inhibitory concentration)

- I don't know (1)
- 4x the MIC (2)
- 8x the MIC (3)
- 10x the MIC (4)
- I use a specific drug level number (5)
- Other (6)

Display This Question:

If Q25 = 5

| 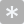 |
| --- |

Q26 **Please list the drug level number you target for the UPPER limit of the CEFEPIME target range (mg/L).**

________________________________________________________________

Display This Question:

If Q25 = 5

| 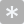 |
| --- |

Q27 **Please list the drug level number you target for the UPPER limit of the PIPERACILLIN target range (mg/L).**

________________________________________________________________

Display This Question:

If Q25 = 5

| 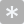 |
| --- |

Q28 **Please list the drug level number you target for the UPPER limit of the MEROPENEM target range (mg/L).**

________________________________________________________________

Display This Question:

If Q25 = 6

| 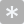 |
| --- |

Q29 **Please list the other UPPER limit of the beta-lactam target range.**

________________________________________________________________

End of Block: Provider – Perceptions

Start of Block: Demographics

| 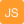 |
| --- |

Q30
Demographics


**Select your primary clinical practice environment.**

- Critical care (1)
- Infectious diseases (2)
- Other (3)

Display This Question:

If Q30 = 1

| 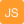 |
| --- |

Q31 **Which one of the following options best describes your critical care practice?**

- Medical (1)
- Non-cardiac surgical (2)
- Mixed medical-surgical (3)
- Cardiac surgical (4)
- Cardiac medical (5)
- Neurointensive care (6)
- Burn (7)
- Other (8)

Display This Question:

If Q30 = 3

Q32 **Please describe your other primary clinical practice environment.**

________________________________________________________________

| 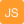 |
| --- |

Q33 **On an average day in the last month, approximately how many patients are assigned to your clinical service?**

- 0 to 5 (1)
- 6 to 10 (2)
- 11 to 15 (3)
- 16 to 20 (4)
- 21 to 25 (5)
- 26 to 30 (6)
- Greater than 30 (7)

| 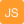 |
| --- |

Q34 **Select your number of years in practice after your terminal clinical training.** (If in training, select 0.)

- 0 years (still in training) (1)
- 1 to 5 years (2)
- 6 to 10 years (3)
- 11 to 20 years (4)
- 21 to 30 years (5)
- Greater than 30 years (6)

End of Block: Demographics

Start of Block: Submit

Q35   THANK YOU FOR COMPLETING THE SURVEY!

 **Please click SUBMIT to record your answers.**

End of Block: Submit
