# Supplementary material for: Provider perspectives on beta-lactam therapeutic drug monitoring programs in the critically ill: a protocol for a multicenter mixed-methods study
Source: Implement Sci Commun. 2021 Mar 24;2:34. doi: 10.1186/s43058-021-00134-9 (PMC7992791; doi:10.1186/s43058-021-00134-9)
Supplement: Supplementary file 3 — Additional file 3. Semi-structured interview guide. [file 43058_2021_134_MOESM3_ESM.docx]

**Semi-structured interview guide**

*Introduction:* Over the last ten years or so, there has been increasing information published about therapeutic drug monitoring for beta-lactams. For the purposes of this discussion, therapeutic drug monitoring or what is sometimes called ‘TDM,’ is the use of drug levels to assess whether a patient is achieving their treatment goals. Commonly in practice TDM is used for other antibiotics like vancomycin and aminoglycosides. Today we are trying to understand its application to beta-lactams such as cephalosporins, penicillins, and carbapenems. Some centers have been performing beta-lactam TDM for years, whereas others have not introduced this practice. We want to talk to clinicians from a variety of backgrounds, environments, and experiences to develop a better understanding of your impressions of beta-lactam therapeutic drug monitoring, specifically for critically ill patients.

*Housekeeping issue*: Before we begin, just want to note that we’d like to record this conversation. We plan to transcribe the tape and review the transcript as a team. The transcript will not include your name but we would like to capture some basic information about you to understand the background and experience that underlies your thoughts. Is that okay with you?

**

1. ***Context.*** To help me have a little the context for your comments, could you tell me briefly about the work you do, the patient population you deal with and how long you have been in practice?

*Probe: If unclear, explicitly ask years of experience total, elsewhere and at current center, job role (e.g. physician, APP, pharmacist), practice type – ICU, infectious diseases, etc.*

*Emphasize that the focus remains on critically ill patients so if they have a mixed practice, hopefully they can reflect on the ICU aspect explicitly*

1. ***Current practice - TDM*:** Can you tell me about how you approach the use of therapeutic drug monitoring or drug level testing for critically ill patients in general?

*Probe: Which drugs, specifically antibiotics (vancomycin, aminoglycosides, beta-lactams), which patients*

Can you give me examples of situations in the critically ill where you feel like this approach works well? Doesn’t work well?

1. ***Current practice – Beta lactams:*** Talking specifically about beta-lactams then, what is your method for dosing and monitoring drugs like cefepime, piperacillin/tazobactam, or meropenem in the critically ill?

*Probe: Patient factors, tools/calculators/software used*

(If not evident from previous answer) On an average day in your practice environment, do you use beta-lactam therapeutic drug monitoring or drug level testing as part of your approach to caring for critically ill patients?

1. (If Yes to 3) ***Barriers/facilitators:*** Can you recall when you first started performing beta-lactam therapeutic drug monitoring?

How did it come on your radar?

How has your understanding and approach to using it evolved over time, or has it?

What do you think the pros- and cons- of beta-lactam therapeutic drug monitoring are?

Were you around for the transition/roll-out of beta-lactam therapeutic drug monitoring in your practice?

If yes – What do you recall about that? What went well? What went poorly?

If no – Think about maybe some other protocol or orderset that rolled out, what factors made that successful? What could have been better?

*Probes: Team dynamics (critical care, ID, pharmacists, lab, etc.), institutional policies/procedures, electronic tools, test turnaround time, calculations, education, familiarity, published guidelines (SCCM, ESICM, etc.)*

There are a number of centers who don’t currently have beta-lactam therapeutic drug monitoring. What are your thoughts on that?

Would you advocate for them getting it?

If so, what would be your recommendations to them as to how to begin understanding it and using it in the critical care environment? I imagine there was a learning curve for you and probably similarly there would be for others.

1. (If No to 3) ***Barriers/facilitators***: What are your thoughts on beta-lactam therapeutic drug monitoring?

What do you think the pros- and cons- of beta-lactam therapeutic drug monitoring are?

We’re interested in implementation of something like this. Think about maybe some other protocol or orderset that rolled out in your practice, what factors made that implementation successful? What could have been better?

*Probes: Team dynamics (critical care, ID, pharmacists, lab, etc.), institutional policies/procedures, electronic tools, test turnaround time, calculations, education, familiarity, published guidelines (SCCM, ESICM, etc.)*

If you draw parallels then, how do you think implementing beta-lactam therapeutic drug monitoring should be approached?

1. ***What else?*** Is there anything we didn’t touch on that you think we should consider as we look at this issue?

(Thank them, plan to share the results and feed-back the findings.)
